# Therapeutic Leukapheresis in a Pediatric Acute Myeloid Leukemia (AML) Crisis as a Critical Life-Saving Adjunctive Therapy: A Case Report

**DOI:** 10.7759/cureus.67874

**Published:** 2024-08-26

**Authors:** Soundharya V, Arthi R, Suresh Kumar I, Hari Haran A, Sahayaraj James

**Affiliations:** 1 Transfusion Medicine, Saveetha Medical College and Hospitals, Saveetha Institute of Medical and Technical Sciences, Saveetha University, Chennai, IND

**Keywords:** leukostasis, tumor lysis syndrome, leukapheresis, acute myeloid leukemia, hyperleukocytosis

## Abstract

Hematological malignancies can present with severe complications, including hyperleukocytosis, which demands immediate intervention due to elevated leukocyte counts that increase blood viscosity and cause microcirculatory abnormalities. This case report highlights the critical role of therapeutic leukapheresis in managing a pediatric patient with acute myeloid leukemia (AML) in crisis. A six-year-old male child presented with symptoms of severe hyperleukocytosis, including high-grade fever, respiratory distress, and a significant leukocyte count (555,820/µL). Immediate interventions included intravenous hydration, antibiotics, steroids, and non-invasive ventilation, followed by therapeutic leukapheresis. The leukapheresis procedure successfully reduced the leukocyte count by 81% without any adverse events. Post-procedure treatment with etoposide further decreased the leukocyte count, leading to symptomatic improvement and stabilization of the patient. The patient was later discharged in stable condition and continued receiving induction-phase chemotherapy. This case underscores the efficacy and necessity of leukapheresis as a life-saving adjunctive therapy in pediatric AML crises, demonstrating its role in rapidly reducing leukocyte counts and preventing life-threatening complications.

## Introduction

Hematological malignancies present with a variety of complications, among which leukocyte count stands as a crucial factor. Hyperleukocytosis is an emergent condition that necessitates immediate therapy [1]. Childhood acute myeloid leukemia (AML) is a clonal disorder marked by the excessive production of abnormal blood cells in the bone marrow, resulting from the malignant transformation of a bone marrow-derived stem cell or progenitor. This leads to the accumulation of immature, nonfunctional myeloid cells, which proliferate in the bone marrow and other organs. To be classified as acute, AML typically requires the bone marrow to contain more than 20% immature leukemic blasts [2]. Elevated leukocyte counts predispose patients to serious complications, primarily due to increased blood viscosity and resultant microcirculatory abnormalities, as well as tumor lysis syndrome (TLS). The rigidity of blast cells in acute leukemia leads to microcirculatory obstruction, increased blood viscosity, hypoxic endothelial injury, and cytokine release, manifesting as pulmonary and neurological symptoms [3].

Leukapheresis is a therapeutic procedure performed to reduce the elevated white blood cell (WBC) count, thereby preventing the complications of leukostasis and hyperviscosity, which can lead to symptomatic hyperleukocytosis. This condition can cause severe complications, such as decreased tissue perfusion, disseminated intravascular coagulation (DIC), intracranial hemorrhage, and respiratory or neurological distress [4]. Using intermittent flow cell separators based on gradient density centrifugation, therapeutic leukocytapheresis is an automated method aimed at depleting WBCs in leukemia patients with leukostasis [3].

Therapeutic leukocytapheresis, as recommended by the American Society of Apheresis (ASFA), is a grade-2B indication for managing leukemia with a WBC count exceeding 100 × 10^9/L and associated leukostasis [5]. Leukapheresis is often combined with cytoreductive agents, reducing the risk of TLS, allowing safe and timely initiation of chemotherapy, thereby mitigating associated risks and improving patient outcomes [6].

In this case report, we explore the critical role of therapeutic leukapheresis as a life-saving adjunctive therapy in a pediatric patient experiencing an acute leukemic crisis, highlighting its effectiveness and necessity in managing severe hyperleukocytosis and its associated complications.

## Case presentation

A six-year-old pediatric patient presented with a one-week history of high-grade fever, vomiting, loss of appetite, cough, cold, and difficulty breathing. Upon examination, the patient exhibited subconjunctival hemorrhage, hepatosplenomegaly, and generalized lymphadenopathy. Due to reduced air entry in the left chest, oxygen support was promptly initiated. Baseline investigations at admission revealed a hemoglobin (Hb) level of 7.3 g/dL, a total leukocyte count (TLC) of 555,820/µL, and a platelet (PLT) count of 74,000/µL. Subsequent peripheral smear, bone marrow aspiration, and biopsy supported the diagnosis of AML-M1 (minimal maturation), with an alarming 97% blasts, 1% myelocytes, 1% promyelocytes, and 1% metamyelocytes. Flow cytometry confirmed the diagnosis of AML-M1 (minimal maturation), showing moderate CD45 expression, medium side scatter with abnormal myeloid blasts, and positive cytoplasmic myeloperoxidase (Cyto MPO) marker. Additionally, cytogenetic testing further identified the presence of NUP98-NSD1 fusion, Fms-like tyrosine kinase 3 (FLT3) mutation, and CCAAT-enhancer binding protein alpha (CEBPA) mutation, indicating AML-M1 type with high-risk cytogenetics.

Immediate treatment included intravenous hydration, antibiotics, and steroids. However, as the patient’s respiratory distress worsened, non-invasive ventilation was initiated. In view of hyperleukocytosis, plans were made for immediate leukapheresis. Informed consent was obtained from the patient’s bystanders for leukapheresis. Pre-procedural investigations, such as serum electrolytes, coagulation profile, liver function tests, and renal function tests, were done and found to be within normal limits. Under strict aseptic precautions, a double-lumen central venous catheter, measuring 16G, was secured into the right femoral vein under ultrasound guidance.

The leukapheresis procedure was carried out using a Com.Tec Fresenius Kabi apheresis instrument. RBC priming was done prior to the initiation of the procedure using leukodepleted packed red blood cells (PRBCs) to prevent hypovolemic complications. In each leukapheresis procedure, a total of 10 liters of blood was processed, which lasted for five to six hours. This required 47 cycles per procedure. Each cycle yielded 10 mL of WBCs (47 cycles × 10 mL = 470 mL). Ultimately, at the end of each procedure, a total of 470 mL of plasma with WBCs was removed.

**Figure 1:**
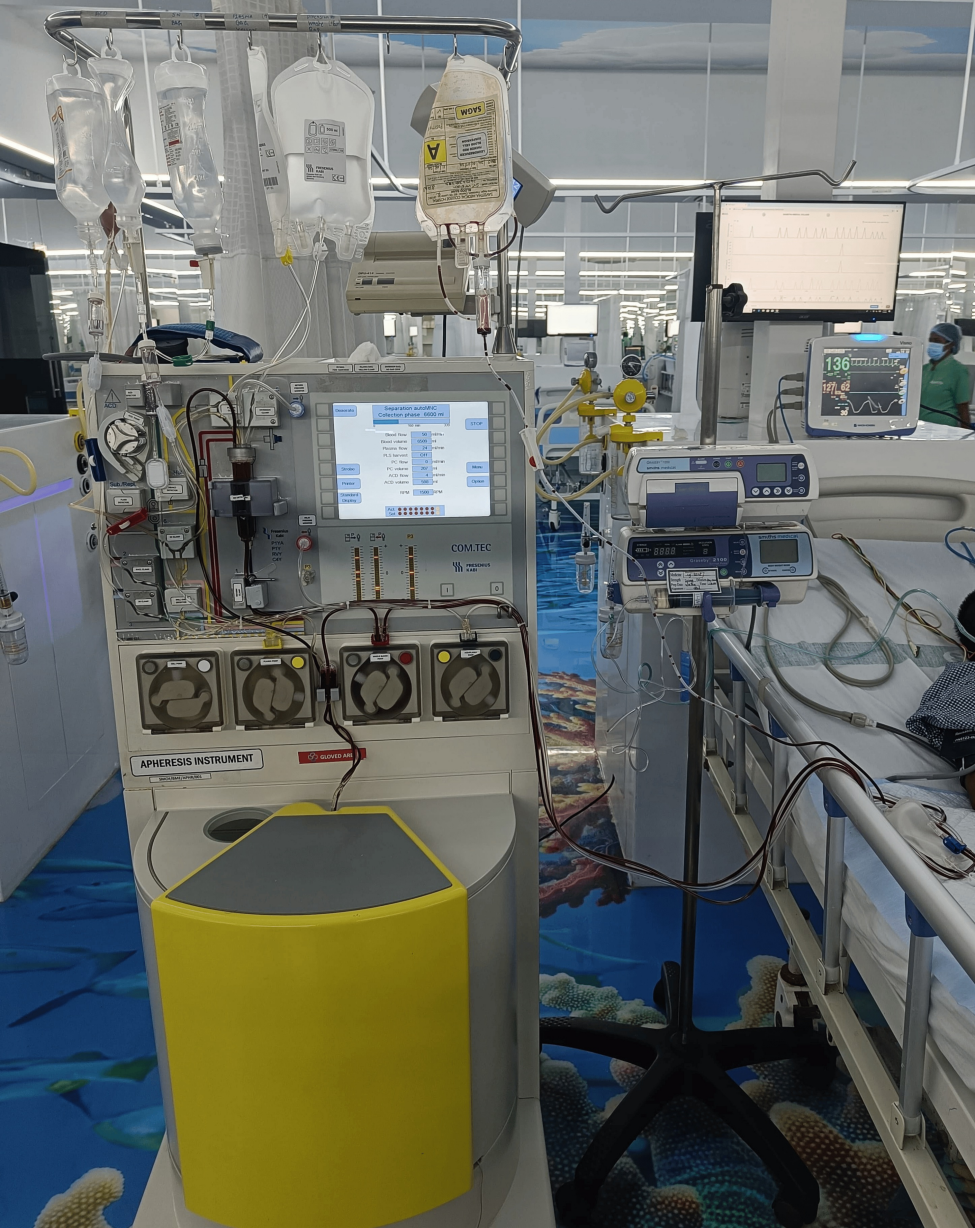
Ongoing leukapheresis procedure using Fresenius Kabi apheresis instrument

Figure 1 illustrates a therapeutic leukapheresis procedure utilizing the Com.Tec Fresenius Kabi apheresis instrument. This process involves selectively separating and removing excess WBCs from the patient's blood to alleviate the risks associated with elevated WBC levels. During the procedure, the patient's blood is drawn into the apheresis machine, where it is centrifuged to isolate and collect the surplus WBCs. Meanwhile, the remaining components of the blood - red blood cells (RBCs), PLTs, and plasma - are safely returned to the patient. This targeted approach helps in managing hyperleukocytosis while maintaining the patient's overall blood composition, thereby reducing potential complications and improving clinical outcomes.

**Figure 2:**
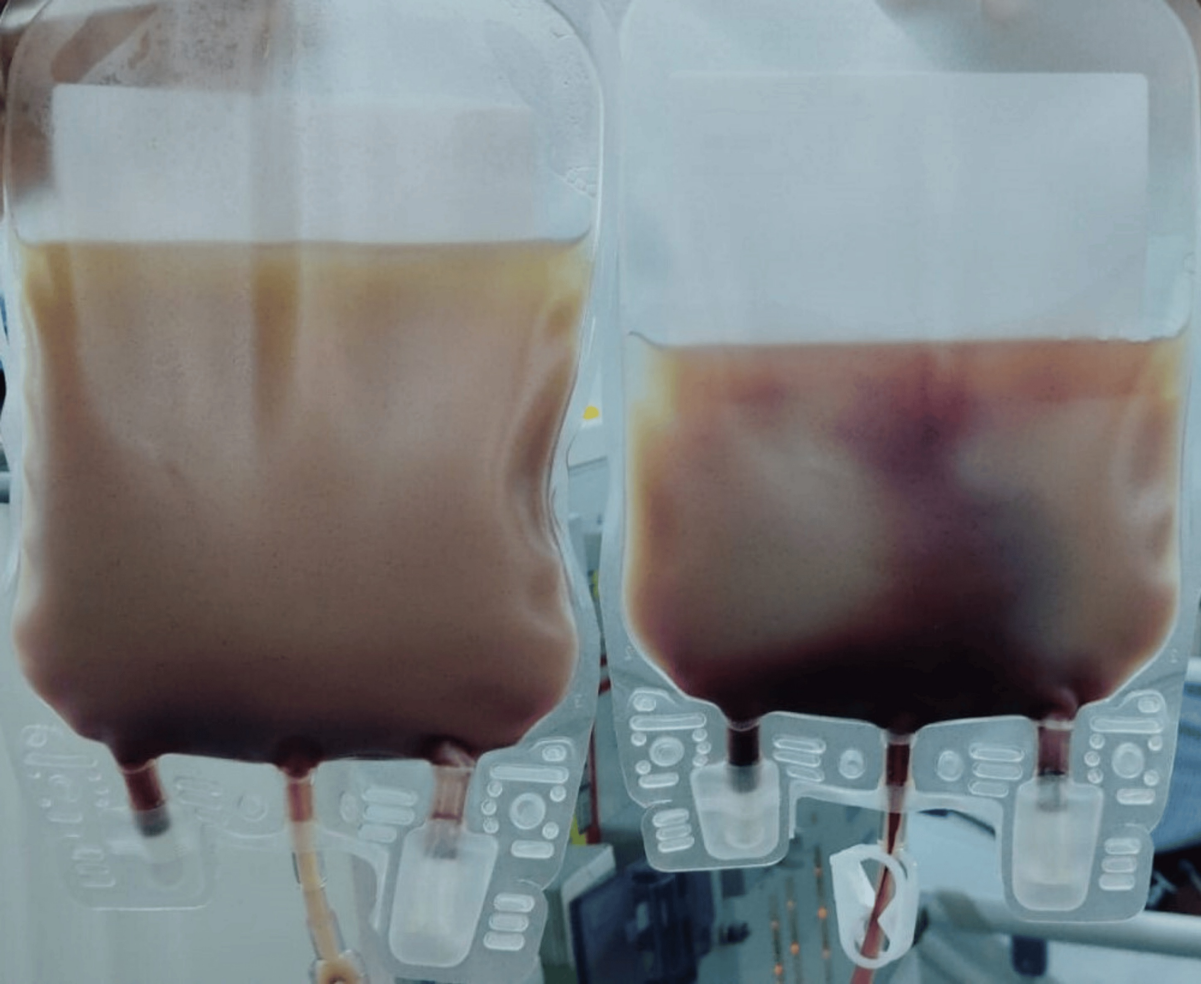
Final product collected post-leukapheresis procedure

Figure 2 depicts the final product obtained after the completion of the apheresis procedure, which consists of a collection of 470 mL of WBCs, along with plasma. This product is the result of the selective removal of excess WBCs from the patient's blood during the therapeutic process.

Normal saline was used to maintain blood volume during the procedure. To prevent acid-citrate-dextrose (ACD)-induced hypocalcemia, the ACD-to-blood ratio was set at 1:14, and 10 mL of 10% intravenous calcium gluconate in 100 mL of 0.9% sodium chloride (NaCl) was administered during the procedure. Also, serum calcium levels and the patient’s vitals were monitored during the procedure. Central line care following the procedure involved a heparin saline flush and sterile gauze packing. Following the first and fourth cycles of leukapheresis, one unit of single donor platelets (SDPs) was transfused, raising the PLT count to 123,000/µL by the end of the procedure.

A total of five consecutive procedures of leukapheresis were successfully performed, reducing the TLC to 108,230/µL (an 81% reduction) without any adverse events or procedure-related complications. Table 1 shows the progression in the clinical course with each successive cycle of leukapheresis. Initially, the patient exhibited severe hyperleukocytosis with a TLC of 555,820 cells/µL, along with anemia (Hb, 7.3 g/dL) and thrombocytopenia (PLT, 74,000 cells/µL). Following each leukapheresis procedure, there was a consistent and significant reduction in TLC, which decreased to 108,230 cells/µL after five procedures. This indicates the procedure’s effectiveness in lowering WBC levels. Serum calcium levels, along with other laboratory parameters, remained stable throughout the procedure, indicating that the treatment was well-tolerated and had no adverse effects on calcium homeostasis.

**Table 1:** Clinical course improvement after each leukapheresis procedure Hb: Hemoglobin; HCT: Hematocrit; TLC: Total leukocyte count; PLT: Platelet

Lab parameters	Pre-procedural counts	Post-leukapheresis procedure 1	Post-leukapheresis procedure 2	Post-leukapheresis procedure 3	Post-leukapheresis procedure 4	Post-leukapheresis procedure 5	Lab parameters (normal range)
Hb (g/dL)	7.3	7.9	8.7	8.9	9.2	9.5	12-16.5
TLC (cells/µL)	555,820	464,460	375,320	284,570	196,240	108,230	4,000-10,000
PLT (cells/µL)	74,000	56,000	99,000	81,000	73,000	123,000	150,000-4,000,000
HCT (%)	31.8	30.6	29.6	28.8	27.6	27.8	40-50
Serum calcium (mg/dL)	8.6	8.3	8.7	8.4	8.5	8.7	8.5-10.5
TLC reduction (cells/µL)	-	91,360	89,140	90,750	88,330	88,010	-

**Figure 3:**
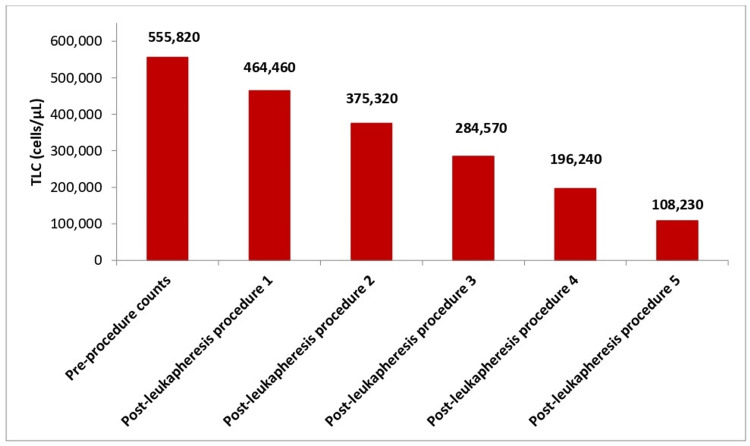
Reduction in the TLC counts after each leukapheresis procedure TLC: Total leukocyte count

Figure 3 presents a graphical representation showing the significant reduction in TLC counts after each leukapheresis procedure, illustrating the progressive decrease from procedure 1 to procedure 5. Post-leukapheresis, the administration of the cytoreductive agent, etoposide, for three days further decreased the TLC to 7,240/µL.

Table 2 demonstrates the pre- and post-leukapheresis outcomes in the patient. Following these treatments, the patient improved symptomatically and was gradually weaned off assisted ventilatory support. By day 10, the patient was hemodynamically stable and subsequently discharged to an oncology center, primarily due to financial constraints, marking a notable improvement from his critical condition at admission.

**Table 2:** Pre- and post-leukapheresis outcomes Hb: Hemoglobin; HCT: Hematocrit; TLC: Total leukocyte count; PLT: Platelet

Lab parameters	Pre-procedure counts	Post-procedure counts	Lab parameters (normal range)
Hb (g/dL)	7.3	9.5	12-16.5
HCT (%)	31.8	27.8	40-50
TLC (cells/µL)	555,820	108,230	4,000-10,000
PLT (cells/µL)	74,000	123,000	150,000-4,000,000
Serum calcium (mg/dL)	8.6	8.7	8.5-10.5
TLC reduction (cells/µL)	-	447,590 (81% reduction)	-

During follow-up, the patient received induction phase chemotherapy using the 7 + 3 regimen, which involved a continuous intravenous infusion of cytarabine for seven days and a daily dose of daunorubicin for three days during the first week of treatment. After the completion of chemotherapy, the patient was discharged and provided with follow-up care.

## Discussion

Hyperleukocytosis, defined by WBC counts exceeding 100,000/µL due to leukemic cell proliferation, significantly increases morbidity and mortality. This condition is most commonly associated with AML, though chronic myeloid leukemia (CML) cases have also been documented [6]. In AML, hyperleukocytosis poses severe risks, such as leukostasis, TLS, and DIC, all of which can lead to early mortality in affected patients [7]. Leukostasis, a condition characterized by multiple organ failure, coagulopathy, and TLS, is a major concern in symptomatic hyperleukocytosis. To prevent leukostasis, leukapheresis is often employed in patients with acute leukemia or hyperleukocytosis-related complications. The ASFA 2023 guidelines categorize therapeutic leukapheresis as a category III, grade 2B indication for hyperleukocytosis [4]. Ganzel et al. recommend leukapheresis when there is significant compromise of the respiratory, central nervous, or renal systems, or when leukocyte or blast counts exceed 100,000/µL. The efficacy of leukapheresis is typically assessed by pre- and post-procedure TLC and the patient's clinical condition. Reports indicate a 20-50% reduction in leukocyte or blast counts after a single leukapheresis session [7].

The role of leukapheresis in improving overall patient outcomes remains debated. While some studies suggest it does not significantly impact early mortality rates, its effectiveness in reducing leukocyte counts and improving clinical symptoms is well-documented. In both AML and CML, initiating chemotherapy in the context of hyperleukocytosis may induce TLS, a life-threatening condition. Hyperleukocytosis-related complications, such as altered blood viscosity and microcirculation obstruction, can lead to dysfunction in organs like the lungs, brain, and liver [8]. The extent of physiological alteration is directly proportional to the TLC. Leukocytapheresis, combined with hydration, can help reduce the risk of TLS and complications related to hyperviscosity, especially in patients with extremely high TLC [9]. This procedure leads to rapid cytoreduction without affecting coagulation parameters [8].

The critical leukocyte count for leukostasis varies with leukemia type. In AML, a leukocyte count as low as 50,000/µL can cause severe symptoms, while CML patients might remain asymptomatic even with counts as high as 500,000/µL due to the differentiated nature of the cells, which tend to sequester in the liver and spleen [2]. Initial treatment typically involves supportive care, with decisions regarding cytoreduction through chemotherapy, hydroxyurea, or leukapheresis based on the patient's clinical status and TLC [10]. Generally, 1.5-2 blood volumes are processed during leukapheresis [11].

In CML with hyperleukocytosis, the condition is usually managed with chemotherapy [10]. However, leukapheresis can rapidly reduce leukocyte counts, serving as a temporizing measure until chemotherapy takes effect, which may take 24-48 hours to achieve similar leukoreduction [12]. Leukapheresis is particularly beneficial in managing complications associated with hyperviscosity and TLS when combined with chemotherapy and supportive treatments, like hydration. Despite the absence of randomized trials for such patients, the safety of leukapheresis generally leads clinicians to proceed with the procedure based on the patient's clinical status and TLC exceeding 100,000/µL [7]. Challenges associated with leukapheresis include potential red cell or PLT loss, necessitating careful monitoring and management of coagulation parameters. There is also a risk of rebound leukocytosis due to the mobilization of sequestered cells, which underscores the need for continued vigilance and possibly repeated sessions [13].

Therapeutic leukapheresis acts as a critical and timely intervention for managing hyperleukocytosis in AML and CML, enabling rapid reduction of leukocyte counts and mitigating complications until definitive chemotherapy can be initiated. However, it requires meticulous monitoring and management to address potential complications and ensure patient safety.

## Conclusions

Leukostasis secondary to hyperleukocytosis is a life-threatening complication of acute leukemia, carrying a poor prognosis due to high morbidity and mortality risks. This case report underscores the urgent need for immediate and aggressive management, including leukapheresis. When promptly administered alongside supportive treatments, like hydration and cytoreductive agents, leukapheresis reduces the risk of TLS, enabling the safe and timely initiation of chemotherapy, thereby improving patient outcomes. As a relatively safe and effective intervention, leukapheresis significantly lowers leukocyte or blast counts, emphasizing its essential role as a life-saving adjunct in AML crises and its importance in the comprehensive management of this severe condition.
